# Misreporting of Patient-Relevant and Study Design Elements in Anesthesiology Randomized Controlled Trials: An Observational Study

**DOI:** 10.3390/medsci13040299

**Published:** 2025-12-03

**Authors:** Igor Vuković, Shelly Melissa Pranić

**Affiliations:** 1 Department of Anesthesiology, Resuscitation and Intensive Care Medicine, General and Veteran Hospital “Hrvatski ponos” Knin, 22300 Knin, Croatia; 2 Department of Public Health, University of Split School of Medicine, 21000 Split, Croatia; shelly.pranic@mefst.hr

**Keywords:** anesthesiology RCTs, ClinicalTrials.gov, trial registration, data reporting, FDAAA

## Abstract

**Background/Objectives**: The quality of trial data reporting within the field of anesthesiology has, to date, been insufficiently examined. This study aims to investigate the consistency of reporting for WHO Data Set Items, trial outcomes, and adverse events between the data reported in ClinicalTrials.gov and the corresponding peer-reviewed publications in a cohort of anesthetic-related randomized controlled trials (RCTs) subject to the FDAAA. **Methods**: In a cross-sectional study, we investigated RCTs performed on 29 drugs in anesthesiology. We examined data reporting for eight categories, including the results and outcome probability measures, adverse events, all-cause mortality, study size, study outcome, study arm, selection criteria, and date of enrollment. We also examined compliance with the ClinicalTrials.gov registration deadline. Using descriptive statistics, we described the reporting reliability in both trial registration and corresponding publication data. Differences in the frequencies of discrepant or inadequate data reporting between selected subgroups were analyzed. **Results**: We identified 258 trials from 2009 to 2022 from ClinicalTrials.gov with corresponding publications. Of these, 28.7% were retrospectively registered. Discrepancies in reporting results occurred in 33.3% of the trials, with serious adverse events in 62.4% and other adverse events in 67.4% of the trials. Primary outcomes were reported much more consistently than secondary ones (77.5% vs. 27.9%). The selection criteria (24%) and enrollment date (29.5%) were the least consistently reported data categories. The only data item with improved reporting over time was all-cause mortality. **Conclusions**: Trial data in anesthesiology clinical trials continue to be misreported. Responsible parties involved in the peer-review process should consider using trial data registration forms as valuable sources for validating the integrity of trial data. Additionally, discrepancies along manuscript progression from submission to publication raise the question about the reliability of both registered and published data as sources for clinical decisions and meta-research.

## 1. Introduction

Ensuring the reliability and credibility of evidence from clinical trials is essential. Accordingly, the quality and transparent reporting of patient-centered and study design elements is required for methodologically sound and ethical research, as reinforced by legislative initiatives [1,2]. In the US, the 2007 Food and Drug Administration Amendment Act (FDAAA) requires the registration of all interventional clinical trials to the ClinicalTrials.gov registry, with the mandatory disclosure of results and adverse events, other than phase 1 trials about Food and Drug Administration (FDA)-regulated drugs, biological products, or devices [3,4]. The FDAAA was revised in 2016, and the Final Rule, effective from 18 January 2017, established additional requirements to facilitate researchers’ reporting of study design elements [5]. Integral to this law is a trial registration deadline of no later than 21 days after the enrolment of the first participant [6]. Accordingly, the International Committee of Medical Journal Editors (ICMJE) enforces a policy [7] requiring the pre-registration of trials in any designated primary registry of the World Health Organization’s (WHO) International Clinical Trials Registry Platform (ICTRP) before publication submission to their member journals. A total of 24 standardized data items created by the WHO are endorsed by the ICMJE, which are the minimum required data for registries in the ICTRP. Despite the requirements set forth by the FDAAA, ICMJE, and WHO to promote the complete reporting of trial data, the underreporting of adverse events and inconsistencies in trial data persist [8,9]. Regarding the compliance with registration deadline policies, recent studies [10,11] showed that at least one-third of randomized controlled trials (RCTs) were registered after the study began. Additionally, other studies [12,13,14] have reported ongoing discrepancies between registry data and publication reports. As the pre-registration and complete reporting of trial data decrease biased reporting in publications, it is surprising that a majority of reviewers still do not use registry data during the peer-review process to check for any discrepant data in manuscripts considered for publication [15].

Highlighting the underreporting of essential data in anesthesiology RCTs is warranted, given the context of the severity of cases and potential complications in this field [16,17]. However, while clinical trials are crucial in improving anesthesiology methods, reporting practices from anesthesiology drug trials are insufficiently studied, with few existing studies pointing out discrepant outcome reporting and signs of publication bias [18,19,20]. Oliveira et al. and Jones et al. both examined the reporting quality of a single or a few selected study design elements in trials published in several of the highest-ranking anesthesiology journals according to impact factors. Furthermore, the selective underreporting of data from anesthesiology trials is likely to have adverse consequences for patients [21]. Consequently, evidence-based decision-making aimed at reducing anesthesia-related morbidity and mortality in routine practice may be hindered by unreliable reporting [22].

Due to the paucity of evidence about the reliability of reporting from anesthesiology clinical trials and the potential impact of the misreporting of patient data, we aimed to investigate the consistency of the reporting of WHO Data Set Items, trial results, and adverse events between the data reported in ClinicalTrials.gov and the corresponding publications in a cohort of anesthetic-related RCTs subject to the FDAAA. Our study is the first to examine a broader range of study design elements, conduct a subgroup analysis, and use a cohort identified through the ClinicalTrials.gov registry itself.

## 2. Methods

### 2.1. Study Design and Eligibility Criteria

This study is a cross-sectional study. We adhered to Strengthening the Reporting of Observational Studies in Epidemiology (STROBE) guidelines for cross-sectional observational studies [23] while preparing the manuscript.

The inclusion criteria were as follows: RCTs (a) having a ClinicalTrials.gov registration number and (b) investigating 29 drugs used in anesthesiology [24]: propofol, thiopental, midazolam, etomidate, ketamine, sevoflurane, isoflurane, desflurane, enflurane, halothane, xenon, nitrous oxide, fentanyl, alfentanyl, sulfentanyl, remifentanyl, morphium, succinylcholine, rocuronium, vecuronium, pancuronium, atracurium, cisastracurium, mivacurium, suggamadex, bupivacaine, levobupivacaine, ropivacaine, and dexmedetomidine.

We excluded trials that were (a) first posted on ClinicalTrials.gov two years after the FDAAA mandate (27 September 2009), allowing a period of two years for trials to publish and post results; (b) last updated on ClinicalTrials.gov after the day of our search, 6 May 2022; (c) non-interventional studies; (d) phase 1 clinical trials; (e) trials that were not designated as completed by the time of the search; (f) trials that are not subject to the FDAAA New Rule; (g) trials that have not disclosed results to ClinicalTrials.gov; (h) trials that did not have a corresponding publication; and (i) trials with published results that were pooled with the results of other trials.

### 2.2. Primary and Secondary Outcomes

Discrepancies and inadequacies in data item reporting were first examined using descriptive statistics. With the awareness of the FDAAA reporting requirements, we selected among the WHO Data Set Items the following data categories that we chose due to their importance in conveying relevant patient and study design information: (1) results and outcome probability measures (reported statistical methods, *p*-values, IQRs, and confidence intervals); (2) adverse events (stratified into serious (SAEs) and other adverse events (OAEs); (3) all-cause mortality; (4) study size; (5) study outcome; (6) study arm; (7) selection criteria; (8) date of enrollment; and (9) frequencies of compliance to the ClinicalTrials.gov trial registration deadline (one month after the study start date). All of the specific cases of misreporting were recorded and presented in tabular format.

Our primary outcome variables were also the frequencies in discrepant reporting between the most recent ClinicalTrials.gov registered version and the corresponding publications for these data categories using the following independent variables: (a) trial funding type (industry or non-industry funding was determined from funding information provided by ClinicalTrials.gov records); (b) whether or not the trial was published in a journal claiming to follow ICMJE recommendations (journals were cross-checked with the official list provided by the ICMJE at the time of data extraction [25]); (c) whether or not the trial complied with ClinicalTrials.gov legislation regarding the deadline for trial registration (information extracted from trials’ corresponding ClinicalTrials.gov entry); (d) whether the trial was conducted by a single research center or was multicentric; and (e) whether the trial belongs to older (stratified into two groups; 2009–2012 and 2013–2016) or more recent (2017–2020) publications in our cohort based on the updated FDAAA legislation in 2017. No sample size calculation or power analysis was necessary.

For our secondary outcome, given the importance of the Final Rule’s implications (see Section 2.4), we conducted a subgroup analysis within our cohort, comparing mortality reporting in trials registered before and after the Final Rule compliance deadline.

We also designed a binary logistic regression model that used the same subgroups (a)–(e) as predictor variables (except we stratified trials by time period in the same way as for the first secondary outcome) to explore the association between reporting discrepancies and the independent variables. For the dependent variable, we selected the existence of a reporting discrepancy in any of the following study data elements: results, SAEs, OAEs, and mortality. Initially, we sought to utilize any reporting discrepancies in the data categories reported in our study. However, since no single RCT in our cohort lacking reporting discrepancies could be identified, we chose to include the data categories that are thematically linked and of special interest for both the clinical application of results and for further meta-research.

### 2.3. Trial and Publication Search and Retrieval

The selection criteria were used as filters in the ClinicalTrials.gov advanced search feature, with the anesthetic drug name as the primary search term. One author (IV) downloaded the search results into a spreadsheet file for subsequent data analysis. National Clinical Trial (NCT) identifier numbers were used to remove duplicates from the search.

ClinicalTrials.gov provides links to associated publications of studies cited in MEDLINE, but, to reduce accessing potentially non-relevant publications that did not report current data from our selected trials [26], we also searched MEDLINE via PubMed using the NCT number from ClinicalTrials.gov along with the PubMed secondary source identification tag [si] (e.g., NCT01068600[si]) for trials that did not contain links to publications. The NCT study identifier was also used as a search query in Google Scholar. Manual searches in PubMed and Google Scholar for publications were also performed using the author’s name and the study title found on ClinicalTrials.gov.

### 2.4. Data Extraction from ClinicalTrials.gov and Publications

Any quantitative or qualitative difference between ClinicalTrials.gov’s most recent available trial entries and the corresponding data in publications was deemed discrepant. Numeric discrepancies were considered to be differences in quantitative data such as a rating scale value or number of patients affected. Qualitative data discrepancies changed the meaning or interpretation of the reported data. Qualitative discrepancies included outcome data or selection criteria presented in one version and omitted in another, data in publications not presented in a comprehensible manner or only graphically, missing adverse events categories in the published or the registered version that are present in the other source, or missing any mention of adverse events. ClinicalTrials.gov added an all-cause mortality field to trial records when the Final Rule was enacted. Hence, trials that were completed prior to this period reported deaths as adverse events or outcomes due to the absence of a designated field to report participant death [27]. Accordingly, we deemed trials completed prior to 18 January 2017 (deadline for compliance with the Final Rule), as reporting participant death when explicitly reported in any part of the Results section in ClinicalTrials.gov. For trials completed on or after 18 January 2017, we assessed the reporting of participant death in the all-cause mortality field and other parts of the Results section in ClinicalTrials.gov. All-cause mortality was deemed as not reported if the trialist did not provide the number of patients affected as explicitly zero or frequencies ≥ 1 either in the all-cause mortality field on ClinicalTrials.gov for trials completed after 18 January 2017 or on ClinicalTrials.gov and in publications as adverse event outcome results or as a part of participant flow data. Similarly, SAEs and OAEs were deemed as reported when explicitly stated as zero or had frequencies ≥ 1 on ClinicalTrials.gov and in publications, e.g., trials that did not list zero in the field were treated as not explicitly reporting zero deaths.

Identifying trials that started before registration in ClinicalTrials.gov was critical to this study design; accordingly, we categorized trials as “starting before registration” and “starting after registration” and used these categories in both outcome and subgroup assignment. ClinicalTrials.gov provides the exact date (MM/DD/YYYY) when the trial was first posted. However, since the registry provides the month and year of the trial start without the specific date, it is not possible to calculate exactly whether or not trialists complied with the 21-day deadline for registration after the enrollment of the first participant [11]. In order to avoid bias while categorizing the trials as starting before or after registration, we chose to allow a one-month reporting period to reduce deeming trials as non-compliant with the registration deadline, similarly to the methodology of Zarin et al. [28] but slightly adapting the tolerance window to our study objectives.

### 2.5. Inter-Observer Reliability

To avoid potential data collector bias from the subjective interpretation of discrepancies between the registered and published data, the reliability of independent extractions of the authors (IV and SP) from a 10% random sample of the eligible RCTs was performed. We obtained a high inter-observer reliability, ranging from the lowest kappa of 0.65 (95% CI 0.43–0.87) for study size and study arm reporting to the highest kappa of 1.00 for study size and mortality reporting. We reached an agreement concerning separate primary and secondary outcome assessments through consensus discussions after achieving a high reliability (kappa = 0.83, 95% CI 0.61–1.00) on the same assessments from the 10% random sample for the outcomes combined. The senior author (SP) reviewed all the remaining extractions through IV. Data extracted from ClinicalTrials.gov were numerically coded according to a previously calibrated coding manual based on consensus discussions between the authors.

### 2.6. Statistical Analysis

We reported descriptive data using frequencies and percentages for categorical variables. To compare the frequencies of discrepant data reporting between the registry and publications, for categorical variables, we performed Chi-square analyses to determine the differences between frequencies. We used Cramer’s V (φc) to measure effect size. We used binary logistic regression analysis to determine the association between any discrepancy in the results, SAEs, OAEs, and mortality data (creating a single dependent variable with a dichotomous (yes/no) value) and independent variables. We examined the relationships in a single model. Goodness of fit and explained variance based on the Chi-square omnibus tests for model coefficients, Homer–Lemeshow tests, Nagelkerke-R2, and Cox and Snell outputs were performed and reported for each of the models. Collinearity diagnostics were made using the variance inflation factor. Regression outputs were reported with odds ratios (ORs) along with 95% confidence intervals (CIs). All analyses were performed using IBM SPSS Statistics for Windows, version 26 (IBM Corp., Chicago, IL, USA). We considered statistical tests with *p*-values below 0.05 as significant.

## 3. Results

### 3.1. Trial Selection and Characteristics

**Figure 1:**
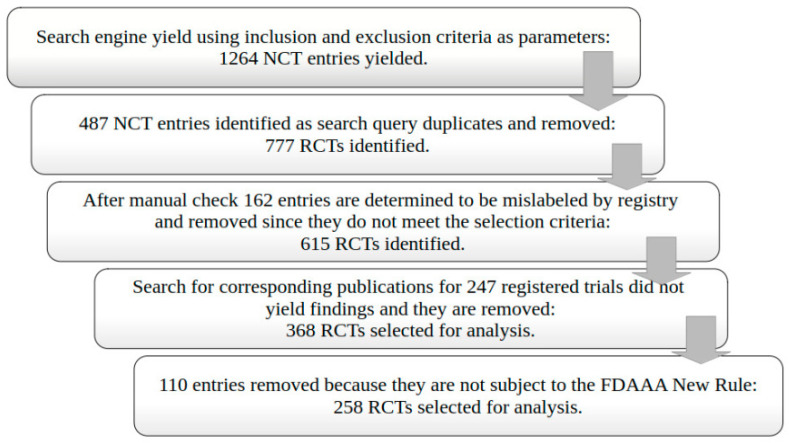
Randomized controlled trial (RCT) selection flowchart.

**Figure 2:**
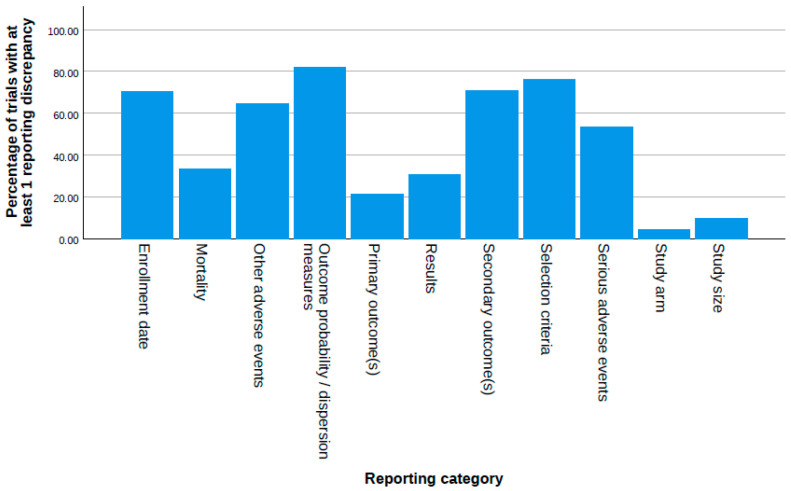
Proportion of trials with reporting discrepancies and/or inadequacies for each data item category examined in our study.

We selected 258 eligible RCTs according to our selection criteria after the ClinicalTrials.gov search and the removal of duplicates (Figure 1). Most trials were phase 4 (63.6%), non-industry funded (79.1%), and had at least one site in the United States (98.8%) (Table 1). Plain bupivacaine was the most frequently studied drug (27.9%), followed by ketamine and (16.3%) and ropivacaine (14.0%). We did not identify trials matching our selection criteria for eight drugs. Overall, the consistency of the reporting of data items in anesthesiology trials was low (Figure 2). In total, 28.7% of trials failed to register within 21 days of starting, a rate that did not change from 2009 to 2020 (Supplementary Table S1).

### 3.2. Discrepancies in Results and Adverse Events Reporting

Reporting discrepancies occurred in approximately 2/3 of the trials for both serious and other adverse events (Table 2). Predominantly, trials did not report the non-occurrence of SAEs in publications, and most trials that reported OAEs reported more categories only in publications.

All-cause mortality reporting was adequately described in 66.7% of the trials. In 29.5% of trials, this registry field was left blank, meaning no value was entered (i.e., not reported as “0”). None of these trials were registered after the Final Rule (Table 3). Mortality reporting was, however, the only data item with improved reporting over time (*p* < 0.001; φc = 0.694 for improvement from the first to third time period; φc = 0.301 for the second to third; see Supplementary Table S1). A direct comparison of trials registered before and after the Final Rule compliance deadline also supports this finding (*p* < 0.001, φc = 0.359).

In total, 66.7% of trial results were reported consistently; however, the majority of trials failed to report *p*-values and statistical methods in the registry (Table 4).

### 3.3. Discrepancies in Study Design Data Reporting

Primary outcomes were reported much more consistently than secondary ones (77.5% vs. 27.9%). More than half of the trials reported secondary outcomes only in the publication (Table 5).

Study size and study arm reporting were the most consistent in our study, 90.3% and 95.7%, respectively. Selection criteria were consistently reported in 24% of trials and study enrollment dates in 29.5% (see Supplementary Tables S2 and S3). Incongruous reporting did not improve in studies that were late to register (see Supplementary Table S1).

### 3.4. Differences in Discrepant Reporting Between Subgroups

Among our subgroup analyses, industry-funded trials were more compliant with trial deadlines (*p* < 0.001, φc = 0.221) and had better OAE reporting (*p* = 0.001, φc = 0.204), but a more inconsistent probability measure (*p* = 0.015, φc = 0.152) reporting (see Supplementary Table S4). Multi-center studies reported similarly, describing OAEs (*p* = 0.001, φc = 0.208) more consistently, and were more compliant with the registration deadline (*p* = 0.04, φc = 0.128), but probability measures were more inconsistent (*p* = 0.001, φc = 0.213). Trials published in ICMJE-compliant journals reported mortality more adequately than trials in ICMJE-non-compliant journals (*p* = 0.017, φc = 0.149).

### 3.5. Secondary Outcome

The data for our binary logistic regression model are presented in Table 6. The logistic regression model was not statistically significant (χ2 = 10.649, *p* = 0.059). The model explained 7.1% of the variance in discrepancy presence (Nagelkerke R2). Trials completed before the Final Rule compliance deadline were 2.7 times more likely to misreport results, adverse events, or mortality data.

## 4. Discussion

Our study showed that the reporting of patient-centered results and study design elements were inconsistent in anesthesiology trials and corresponding publications. The misreporting of essential data from clinical trials corroborates the findings from previous investigations in other fields of medicine [29,30]. Our study extends these findings to show discrepant and inadequate patient-relevant and study design data reporting in a large sample of anesthesiology RCTs. These results emphasize the importance of peer reviewers and editorial staff comparing registration and publication at the journal level.

Discrepantly reported results and key study elements raise further questions regarding which source is more reliable and closer to accurate study data.

Our study identified that, except for mortality reporting, the reporting of study design and patient-related data from the anesthesiology trials did not improve over time, which is a novel finding. In contrast, a study on the temporal association of trial data reporting and FDAAA legislation showed significant improvements in reporting for almost all data item categories for the 2010–2014 and 2017–2021 time windows [31]. We divided the trials in the current study into similar time windows, and the lack of differences in reporting before and after the FDAAA could be due to differences in the trial subject area or variable reporting practices in other medical disciplines compared to anesthesiology.

It remains pertinent that registered trial data be evaluated for completeness to help prevent discrepancies in registries and subsequent publications. Given that publication is recognized as the ultimate endpoint of any trial, some researchers may view trial registration solely as an administrative requirement. In that case, trialists would not precisely update the data after data entry in a registry so that their submitted paper reporting the trial’s data would be eligible for publication in an ICMJE journal. This could explain why many trialists did not report mortality or adverse event data in the respective fields. Accordingly, we observed that the Final Rule effectively enforced the reporting of all-cause mortality, as it was the only data item with significantly improved reporting over time in our study. We observed differences that could also be due to data entry errors, especially if different individuals are responsible for trial data reporting in registries and publications. Intentional data modification is also possible.

The inconsistencies observed for both results and adverse event reporting in this study potentially hinder the use of ClinicalTrials.gov as a meta-research source. The presence of data in the wrong registry fields may also increase the potential for bias when using automated data extraction or data mining to retrieve data, as described in a previous study [32]. Furthermore, even though the results in this study were reported more consistently than other study elements, a third of the trials had some measure of misreported essential data. Further research is needed to determine whether the discrepancies in results can be attributed to publication bias favoring positive results.

The high frequency of selection criteria discrepancies (76.4%) is especially worrisome in view of possible biased research design. Our finding that studies that were late to register did not report selection criteria more consistently suggests that authors do not engage in deliberate selective data reporting, but do not perceive trial registration as a serious process from which data could be further used. Moreover, one would assume that late-registered trials had a higher frequency of adequate sample size reporting, since they were registered later. Paradoxically, trials in this study that met the registration deadline reported sample sizes more consistently, perhaps owing to better reporting practices.

More industry-funded trials in our study posted results within one year and were more precise with other adverse event reporting than non-industry-funded trials. Perhaps industry funding allows trialists to be more attentive to adverse event reporting, or industry policies require a stricter adherence to protocols for adequate compliance with registration legislation. Accordingly, a previous study also found that industry-funded trials were prospectively registered more frequently [33].

Our study has some limitations. Due to its design, our data depends heavily on the ClinicalTrials.gov database and search algorithm. Trials that were finished in reality and published may have been undetected by our search if the authors had not reported the trial as “completed” in the registry and had not disclosed the results. A previous study found that 29.5% of trials failed to disclose results even four years after completion, while those that did were frequently delinquent in reporting within one year [34]. Jones and colleagues showed that some completed RCTs purposely left the trial’s status as “ongoing” indefinitely to avoid result disclosure [35]. Additionally, our initial screening to select RCTs revealed that some were mislabeled as anesthetic trials in the registry but involved other drugs, highlighting a source of potential selection bias. As previously mentioned, it was not possible to determine the exact trial registration date if only the month and year were available on ClinicalTrials.gov, so we allowed a one-month leeway to avoid deeming a trial as late to register. However, the actual number of trials that were late to register could be higher than in our study. Additionally, due to the unpredictability of the time required to publish articles, it is possible that authors had insufficient time to revise peer-reviewed submissions. Our subgroup analysis included testing for a wide spectrum of hypotheses; however, since we did not adjust for multiple comparisons in our analysis, it is possible that some statistically significant findings are in fact random occurrences. Additionally, other factors we did not account for could have influenced the reporting of data in ClinicalTrials.gov after the final rule of the FDAAA. Lastly, the omnibus test for the logistical regression model was not statistically significant; thus, caution should be exercised when interpreting the relationship between the outcome and predictor variables. The results of the present study can be generalized to other completed ClinicalTrials.gov trial records with similar data and results received after the FDAAA mandate.

Our trial showed that anesthesiology RCTs did not improve in reporting results and other trial data consistently from the implementation of the FDAAA legislation. Peer reviewers, as well as medical journal editors, should routinely adopt discrepancy-seeking in manuscripts with trial data using ClinicalTrials.gov during the review process. If reporting consistency is linked to the publication of trial data, effective reviewer training may play a role in raising the quality of data reporting and registration compliance, although more evidence is needed regarding the effectiveness of peer reviewer training [36,37].

Authors could also be encouraged to maintain data integrity between sources by following the reporting guidelines available on the EQUATOR Network or the requirements set by study sponsors. A recent initiative among journals is the introduction of a requirement for uploading databases and other raw data to online repositories, aimed at enhancing the transparency of data reporting [38]. Researchers conducting meta-analyses could benefit from an increased awareness of possible data discrepancies in clinical trial registries. Ultimately, practicing anesthesiologists as the end users of every trial publication need to be informed about the additional ways of evaluating data reporting quality. Our results could provide improved insight into the transparency of clinical trial reporting in anesthesiology and highlight sources of publication bias where certain trial data are altered. These practices can influence the composition of professional medical practice guidelines and clinicians’ drug prescription decisions.

## Figures and Tables

**Table 1:** Study characteristics.

**Total Number of Trials: 258**			
**Trial Corresponding Publications by Year (n, %)**			
2009	3 (1.2%)	2015	44 (17.1%)
2010	15 (5.8%)	2016	35 (13.6%)
2011	20 (7.8%)	2017	40 (15.5%)
2012	26 (10.1%)	2018	15 (5.8%)
2013	26 (10.1%)	2019	6 (2.3%)
2014	27 (10.5%)	2020	1 (0.4%)
**Study Size**			
Median		Range	
80		1554	
**Number of Trial Study Arms**			
Median		Range	
2.00		6.00	
**Number of Trial Primary Outcomes**			
Median		Range	
1.00		8.00	
**Study Funding Source (n)**			
Industry		54	
Non-industry		204	
**Trial Location**			
United States of America:	255	Turkey	2
Austria: Belgium Denmark Germany	4	Australia Czechia Finland Lithuania South Korea Taiwan	1
Spain	3		
**Trial Clinical Phase (n, %)**			
Phase 1|Phase 2		4 (1.6%)	
Phase 2		35 (13.6%)	
Phase 2|Phase 3		12 (4.7%)	
Phase 3		43 (16.7%)	
Phase 4		164 (63.6%)	
**Trials by Studied Anesthetic (n, %)**			
Bupivacaine	72 (27.9%)	Desflurane	5 (1.9%)
Ketamine	42 (16.3%)	Remifentanyl Sulfentanyl N2O	4 (1.6%)
Ropivacaine	36 (14.0%)		
Morphine	33 (12.8%)		
Liposomal bupivacaine	33 (12.8%)	Vecuronium	3 (1.2%)
Fentanyl Midazolam	28 (10.9%)	Alfentanyl Cisatracurium Etomidate Isoflurane	2 (0.8%)
Propofol	26 (10.1%)		
Suggamadex	12 (4.7%)		
Sevoflurane	8 (3.1%)	Succinylcholine	1 (0.4%)
Rocuronium	6 (2.3%)		

**Table 2:** Types and frequencies of adverse event reporting discrepancies between Clinicaltrials.gov registered version and publication of the same trial.

OAE **	SAE *	Study Adverse Events Reporting Discrepancies, n (%)
84 (32.6%)	97 (37.6%)	Consistent reporting
4 (1.6%)	3 (1.2%)	Consistent reporting but data provided in wrong data input field in Clinicaltrials.gov ^a^
13 (5%)	4 (1.6%)	Quantity greater in the registry
12 (4.7%)	2 (0.8%)	Quantity greater in the publication
50 (19.4%)	20 (7.8%)	At least one adverse event description reported only in the registry
3 (1.2%)	0 (0%)	At least one adverse event reported only in the registry but using surrogate measures with no quantification provided
13 (5%)	0 (0%)	Higher threshold percentages reported in the publication than in the registry
58 (22.5%)	33 (12.8%)	At least one adverse event category reported only in the publication
7 (2.7%)	1 (0.4%)	Textual descriptions only that are absent in ClinicalTrials.gov
1 (0.4%)	0 (0%)	Adverse event data presented inconsistently making it impossible to compare ^b^
45 (17.4%)	84 (32.6%)	Adverse event data not stated at all in the publication while reported as “0” in the registry
3 (1.2%)	20 (7.8%)	No explicit statement in the publication about the reported adverse events being all that occurred ^a^
1 (0.4%)	3 (1.2%)	OAE misclassified as SAE/SAE misclassified as OAE in the registry ^a^

^a^ Despite inadequacies, not counted as discrepant reporting input in the statistical analysis. ^b^ Incomparable due to adverse events presented in a graph or figure in publications while specific values were described in Clinicaltrials.gov. Abbreviations: * SAE, serious adverse event; ** OAE, other adverse event.

**Table 3:** Types and frequencies of mortality reporting discrepancies between ClinicalTrials.gov registered version and publication of the same trial.

All-Cause Mortality Reporting Discrepancies, n (%)	
172 (66.7%)	Consistent reporting
1 (0.4%)	Lower frequency in publication
2 (0.8%)	Lower frequency in registry
3 (1.2%)	Reported in registry and omitted completely in publication
3 (1.2%)	Reported in publication and omitted completely in registry
1 (0.4%)	Reported in the SAE * table in ClinicalTrials.gov
76 (29.5%)	Not reported in ClinicalTrials.gov **

* Abbreviations: SAE, serious adverse event. ** Trialists left blank fields for all-cause mortality reporting in the registry. All of the instances where trials are not required to be New Rule compliant.

**Table 4:** Types and frequencies of study primary and secondary outcome reporting discrepancies between ClinicalTrials.gov registered version and publication of the same trial.

Study Outcome Reporting Discrepancies, n (%) *	
200 (77.5%)	Primary outcome reported consistently
17 (6.6%)	Primary outcome detail reported inconsistently (timeframe or method of evaluation)
12 (4.7%)	Timeframe
7 (2.7%)	Methods
9 (3.5%)	Primary outcome present solely in the registry
16 (6.2%)	Primary outcome present solely in the publication
13 (5%)	Registered secondary outcome promoted to primary in publication
9 (3.5%)	Registered primary outcome demoted to secondary in publication
7 (2.7%)	Unclear whether newly added outcome in publication is primary or secondary
3 (1.2%)	Publication did not explicitly differentiate outcomes as primary or secondary ^a^
72 (27.9%)	Secondary outcome reported consistently
13 (5%)	Secondary outcome detail reported inconsistently (timeframe, method of evaluation, etc.)
11 (4.3%)	Timeframe
4 (1.6%)	Methods
77 (29.9%)	Secondary outcome present solely in the registry
133 (51.6%)	Secondary outcome present solely in the publication

^a^ Despite inadequacy reported, not deemed as a discrepancy input in the statistical analysis. * A single RCT could have multiple outcome reporting discrepancies.

**Table 5:** Types and frequencies of results and result probability measure reporting discrepancies between ClinicalTrials.gov registered version and publication of the same trial.

**Study Results Reporting Discrepancies Between ClinicalTrials.gov and Publications, n (%)**	
172 (66.7%)	Consistent reporting
7 (2.7%)	Registered primary outcome not designated as primary in the publication ^a^
22 (8.5%)	Quantities of assessments greater in the publication
12 (4.7%)	Quantities of assessments greater in the registry
1 (0.4%)	Assessed parameters reported only in the registry
1 (0.4%)	Results data expanded in publication with more timepoints for collected data
1 (0.4%)	Results data reduced in publication with less timepoints for collected data
17 (6.6%)	Results data presented inconsistently making it impossible to compare ^b^
4 (1.6%)	Results presented with different method or timeframe for outcome assessment
8 (3.1%)	Quantities of assessments differ in either study arm
5 (1.9%)	Incomparable because outcomes were completely different between sources
1 (0.4%)	Results data swapped between two study arms
1 (0.4%)	Difference in measure of central tendency between sources
1 (0.4%)	Difference in measures of dispersion between sources
Study Result Probability Measure Reporting Discrepancies, n (%) *	
47 (18.2%)	Consistent reporting
179 (69.4%)	*p*-value and/or statistical method not presented in registry
1 (0.4%)	*p*-value and/or statistical method presented in registry but not in publication
7 (2.7%)	Statistical method discrepancy
2 (0.8%)	Statistical method discrepancy, but statistical measure (*p*-value) numerically the same
10 (3.9%)	Probability measure (*p*-value or CI ^†^) discrepancy
2 (0.8%)	Discrepant statistical method and probability measure
2 (0.8%)	Either source presented the *p*-value or CI
1 (0.4%)	*p*-value possibly incorrectly rounded up in the publication
2 (0.8%)	Probability measures (*p*-value or CI) differ due to quantitative results reporting discrepancy
1 (0.4%)	Probability measures (*p*-value or CI) differ due to qualitative results reporting discrepancy (method of evaluation and timeframe are changed in publication)

^a^ For the purposes of statistical evaluation, these discrepancies were reported as outcome discrepancies (see Table 5), not determined as a result reporting discrepancy input in the statistical analysis. ^b^ Incomparable due to adverse events presented in a graph or figure in publications, while specific values were described in ClinicalTrials.gov. * Percentages have been rounded and do not total 100. ^†^ CI, confidence interval.

**Table 6:** Binary logistic regression analysis results for testing the chosen subgroups in our study for the presence of any discrepancy in the following data reporting categories: results, adverse events (both serious and other), and mortality.

	**95% CI ^d^ for OR**							
**VIF ^e^**	**Upper Bound**	**Lower Bound**	**OR ^c^**	***p*-Value**	**Df ^b^**	**Std. Error**	**B ^a^**	**Predictor**
1.006	5.569	1.297	2.688	0.008	1.0	0.372	0.989	Time period
1.383	2.143	0.216	0.681	0.511	1.0	0.585	−0.384	Funding source
1.004	2.298	0.530	1.104	0.791	1.0	0.374	0.099	ICMJE member
1.341	1.903	0.119	0.476	0.294	1.0	0.707	−0.742	Multicenter trial
1.053	1.712	0.340	0.763	0.511	1.0	0.413	−0.271	Late to register
**Nagelkerke R-** **Squared**		**Cox and Snell R** **Square**		**Hosmer and Lemeshow Test**			**Omnibus Tests of Model Coefficients**	
0.071		0.040		***p*-value**	**χ^2^**		***p*-value**	**χ^2^**
				0.586	4.675		0.059	10.649

^a^ B—estimated binary logistic regression coefficients for the models. ^b^ Df—Degrees of freedom. ^c^ OR—Odds ratio. ^d^ CI—Confidence interval. ^e^ VIF—Variance inflation factor.

## Data Availability

The data presented in this study are openly available at Open Science Framework website: https://osf.io/fqad7/?view_only=976e69bdb7a14722befb3d22ae1fd1e1 (accessed on 30 September 2025).

## Supplementary Materials

The following supporting information can be downloaded at https://www.mdpi.com/article/10.3390/medsci13040299/s1. Supplementary Table S1: Association between trial completion date and discrepant trial data reporting between trial data on ClinicalTrials.gov and in publications of the same trial., Supplementary Table S2: Types and frequencies of eligibility criteria reporting discrepancies between ClinicalTrials.gov registered version and publication of the same trial, Supplementary Table S3: Types and frequencies of enrollment date reporting discrepancies between ClinicalTrials.gov registered version and publication of the same trial, Supplementary Table S4: Association between trial subgroups (trial funding source, adherence to the ClinicalTrials.gov registration deadline, single vs multicentric trials and publication in a ICMJE-compliant journal) and discrepant trial data reporting between data registered on ClinicalTrials.gov and in publications of the same trial.

## Abbreviations

The following abbreviations are used in this manuscript:

FDAAA	Food and Drug Administration Amendment Act
ICMJE	International Committee of Medical Journal Editors
WHO	World Health Organization
ICTRP	International Clinical Trials Registry Platform
RCT	Randomized controlled trial
STROBE	Strengthening the Reporting of Observational Studies in Epidemiology
SAE	Serious adverse event
OAE	Other advese event
