# Supplementary material for: Misreporting of Patient-Relevant and Study Design Elements in Anesthesiology Randomized Controlled Trials: An Observational Study
Source: Med Sci (Basel). 2025 Dec 3;13(4):299. doi: 10.3390/medsci13040299 (PMC12735231; doi:10.3390/medsci13040299)
Supplement: Supplementary file 1 [file medsci-13-00299-s001.zip › medsci-4006082-supplementary.pdf]

Supplementary Table S1. Association between trial completion date and discrepant trial data reporting between trial data on ClinicalTrials.gov and in publications of the same trial.

Reporting category	Reporting consistency	A: RCTs[b] from 2009-2012 timeperiod (n=64)	B: RCTs from 2013-2016 timperiod (n=132)	C: RCTs from 2017-2020 timeperiod (n=62)	p value [a] for comparison of columns A and C	p value for comparison of columns B and C
Results	consistent	43	92	44	0.646	0.857
	discrepant	21	40	18		
Outcome probability / dispersion measures	consistent	17	20	10	0.154	0.861
	discrepant	47	112	52		
SAE [c]	consistent	23	71	26	0.490	0.124
	discrepant	41	61	36		
OAE [d]	consistent	15	53	24	0.064	0.848
	discrepant	49	79	38		
Mortality	consistent	19	93	60	**0.000**	**0.000**
	discrepant	45	39	2		
Study size	consistent	60	120	53	0.127	0.257
	discrepant	4	12	9		
Study primary outcome(s)	consistent	46	104	53	0.063	0.268
	discrepant	18	28	9		
Study secondary outcome(s)	consistent	15	37	23	0.095	0.203
	discrepant	49	95	39		
Selection criteria	consistent	13	30	19	0.183	0.237
	discrepant	51	102	43		

Study arm	consistent	64	124	59	0.075	0.731
	discrepant	0	8	3		
Enrollment date	consistent	17	35	24	0.146	0.085
	discrepant	47	97	38		
Registration deadline	compliant	44	96	44	0.786	0.799
	late	20	36	18		

[a] We used chi-squared analysis and considered P values less than 0.05 to be significant. [b] RCT-randomized controlled trial. [c] SAE - Serious adverse events. [d] OAE - Other adverse events.

Supplementary Table S2. Types and frequencies of eligibility criteria reporting discrepancies between Clinicaltrials.gov registered version and publication of the same trial.

**Eligibility criteria reporting discrepancy, n (%)**	
Selection criteria reported consistently	62 (24 %)
Minimum or maximum values in specific criterion range changed	51 (19.8%)
Selection criteria is present only in registry	137 (53.1 %)
Selection criteria is present only in publication	105 (41 %)

Supplementary Table S3. Types and frequencies of enrollment date reporting discrepancies between Clinicaltrials.gov registered version and publication of the same trial.

**Study enrollment date reporting discrepancies, n (%)**	
Consistent reporting	76 (29.5 %)
Consistent reporting but data provided in wrong data field in ClinicalTrials.gov	1 (0.4 %)
Mismatching date between sources	130 (50.4 %)
Omitted in publication	51 (19.8 %)

Supplementary Table S4. Association between trial subgroups (trial funding source, adherence to the ClinicalTrials.gov registration deadline, single vs. multicenter trials and publication in a ICMJE [a] - compliant journal) and discrepant trial data reporting between data registered on ClinicalTrials.gov and in publications of the same trial.

Reporting category	Reporting consistency	Industry funded trials (N=54)	Non-industry funded trials (N=204)	p value [b]	Compliant with registration deadline (N=184)	Trials late to register (N=74)	p value	Single-center trials (N=214)	Multi-center trials (N=44)	p value	ICMJE journal (N=92)	Not an ICMJE journal (N=166)	p value
Results	consistent	34	145	0.250	130	49	0.484	150	29	0.583	65	114	0.741
	discrepant	20	59		54	25		64	15		27	52	
Outcome probability / dispersion measures	consistent	16	31	**0.015**	37	10	0.215	31	16	**0.001**	15	32	0.553
	discrepant	38	173		147	64		183	28		77	134	
SAE [d]	consistent	25	95	0.972	84	36	0.663	99	21	0.859	45	75	0.565
	discrepant	29	109		100	38		115	23		47	91	
OAE [e]	consistent	9	83	**0.001**	66	26	0.911	86	6	**0.001**	35	57	0.552
	discrepant	45	121		118	48		128	38		57	109	
Mortality	consistent	38	134	0.516	127	45	0.206	141	31	0.558	70	102	**0.017**
	discrepant	16	70		57	29		73	13		22	64	
Study size	consistent	45	188	0.051	170	63	0.075	194	39	0.680	87	146	0.085
	discrepant	9	16		14	11		20	5		5	20	
Study primary outcome(s)	consistent	44	159	0.572	151	52	**0.036**	168	35	0.878	76	127	0.252
	discrepant	10	45		33	22		46	9		16	39	
Study	consistent	22	53	**0.034**	54	21	0.877	58	17	0.125	29	46	0.518

secondary outcome	discrepant	32	151		130	53		156	27		63	120	
Selection criteria	consistent	11	51	0.479	50	12	0.062	53	9	0.542	24	38	0.565
	discrepant	43	153		134	62		161	35		68	128	
Study arm	consistent	53	194	0.324	177	70	0.565	205	42	0.919	90	157	0.216
	discrepant	1	10		7	4		9	2		2	9	
Enrollment date	consistent	15	61	0.761	55	21	0.809	62	14	0.706	28	48	0.798
	discrepant	39	143		129	53		152	30		64	118	
Registration deadline	compliant	49	135	**0.000**	N/A	N/A	N/A	147	37	**0.040**	68	116	0.493
	late	5	69		N/A	N/A		67	7		24	50	

a ICMJE – International Committee of Medical Journal Editors. b We used chi-squared analysis and considered P values less than 0.05 to be significant. c SAE - Serious adverse events. d OAE - Other adverse events.
